# Effect of GLPG1205, a GPR84 Modulator, on CYP2C9, CYP2C19, and CYP1A2 Enzymes: *In Vitro* and Phase 1 Studies

**DOI:** 10.1002/cpdd.956

**Published:** 2021-05-06

**Authors:** Julie Desrivot, Tim Van Kaem, Lisa Allamassey, Eric Helmer

**Affiliations:** ^1^ Galapagos Romainville France; ^2^ Galapagos Mechelen Belgium; ^3^ Alten Belgium Brussels Belgium; ^4^ Galapagos Biotech Ltd Cambridge UK

**Keywords:** drug‐drug interaction, cytochrome P450, idiopathic pulmonary fibrosis, GLPG1205

## Abstract

GLPG1205 is a novel agent being investigated for the treatment of idiopathic pulmonary fibrosis. GLPG1205 may be concomitantly administered with pirfenidone in future clinical development; therefore, the potential for GLPG1205 to interact with enzymes involved in the metabolism of pirfenidone (cytochrome P450 [CYP] 1A2, CYP2C9, 2C19) was evaluated. In vitro experiments indicated weak inhibition of CYP1A2 and moderate but reversible inhibition of CYP2C9 and CYP2C19 by GLPG1205. A phase 1 randomized, double‐blind crossover study in 14 healthy males (NCT02623296) evaluated the effect of GLPG1205 100 mg or placebo (once daily for 12 days) on the single‐dose pharmacokinetics of a cocktail of CYP1A2, CYP2C9, and CYP2C19 substrates (coadministered on day 13). GLPG1205 had no effect on the exposure of CYP2C9 and CYP1A2 substrates or metabolites; however, a trend toward increased omeprazole (CYP2C19 substrate) exposure was observed. Although considered not clinically relevant, GLPG1205 increased the elimination rate of 5‐hydroxyomeprazole (CYP2C19 metabolite) 1.16‐fold versus placebo. GLPG1205 had no effect on the elimination of all other substrates or metabolites. GLPG1205 had a favorable safety and tolerability profile. In conclusion, GLPG1205 100 mg once daily does not interact with CYP2C9, CYP2C19, or CYP1A2 to a clinically relevant extent and may be administered concomitantly with drugs metabolized by these enzymes.

GPR84 is a G protein‐coupled receptor that is activated by medium‐chain fatty acids and is primarily expressed on a number of immune cells such as leukocytes, macrophages, and monocytes.^1^, ^2^, ^3^ By stimulating fibrosis, GPR84 may have a deleterious role in fibrotic disease, including pulmonary fibrosis.^2^ GLPG1205 (9‐cyclopropylethynyl‐2‐[(S)‐1‐(1,4)dioxan‐2‐ylmethoxy]‐6,7‐dihydropyrimido[6,1‐a]isoquinolin‐4‐one; compound code G321605; Supplemental Figure 1) is a novel modulator of GPR84 currently under development as a therapy for idiopathic pulmonary fibrosis (IPF). A first‐in‐human study (NCT01887106) with healthy subjects showed that GLPG1205 had an acceptable safety and tolerability profile, with a maximum tolerated dose of 100 mg once a day.^4^ In healthy male subjects, GLPG1205 was absorbed with a median time to maximum plasma concentration (t_max_) of 4 hours. After once‐daily oral dosing for 14 days, a steady‐state GLPG1205 plasma concentration was reached by day 10, and the elimination half‐life was on average 88.6 hours.^4^ GLPG1205 50 and 100 mg are currently under phase 2 clinical investigation.

Pirfenidone and nintedanib are currently the only 2 pharmacological options approved for the treatment of IPF.^5^ In future clinical trials, GLPG1205 may be concomitantly administered with pirfenidone and nintedanib; therefore, it is important to investigate the potential for GLPG1205 to interact with enzymes involved in the metabolism of each drug. The mechanism of action of pirfenidone is incompletely understood; however, data have shown that pirfenidone attenuates fibroblast proliferation and the production of fibrosis‐associated proteins and cytokines and increases biosynthesis and accumulation of extracellular matrix in response to cytokine growth factors such as transforming growth factor‐beta and platelet‐derived growth factor.^6^ Pirfenidone is mainly metabolized by cytochrome P450 (CYP) 1A2 and, to a lesser degree, by CYP2C9 and CYP2C19^7^, ^8^; therefore, agents that induce or inhibit these enzymes have the potential to adversely affect exposure to pirfenidone. Nintedanib is a small‐molecule tyrosine kinase inhibitor including the receptors platelet‐derived growth factor receptor α and β, fibroblast growth factor receptor 1‐3, and vascular endothelial growth factor receptor 1‐3.^9^ Nintedanib is primarily metabolized via hydrolytic ester cleavage and only a minor extent of nintedanib biotransformation involves CYP pathways, predominantly CYP3A4.^10^, ^11^ A first‐in‐human study has shown that GLPG1205 did not induce CYP3A4 based on evaluation of the cortisol:6β‐hydroxycortisol ratio in urine^4^, ^12^; therefore, the current studies focused on potential for interactions with pirfenidone, which may be more complex given the involvement of multiple CYP450 enzymes.

The aims of the studies presented in this article were to assess the potential for GLPG1205 to induce/inhibit CYP2C9, CYP2C19, and CYP1A2 in vitro and to clinically assess the effect of multiple doses of GLPG1205 on the pharmacokinetics (PK) of a validated cocktail of substrates^13^ for these enzymes.

## Methods

### In Vitro Studies

Full details of the methods used in both in vitro studies are detailed in the Supplemental Methods. A brief summary is provided below.

#### In Vitro Assessment of CYP1A2 Induction

Solubility and cytotoxicity experiments were performed prior to incubation of GLPG1205 with cells.

GLPG1205 was incubated with monolayer cultures of fresh human hepatocytes from 3 individual donors for 24 hours (messenger ribonucleic acid [mRNA] analysis) or 48 hours (enzyme activity) at 6 different concentrations: 0.5, 1, 5, 10, 50, and 100 μM (0.19, 0.38, 1.89, 3.78, 18.9, and 37.8 μg/mL, respectively). All incubations contained 0.4% dimethylsulfoxide (DMSO). Incubation with 50 μM omeprazole containing 0.1% DMSO was used as a positive control. Incubations with 0.1% and 0.4% DMSO alone were used as negative controls.

Following a 24‐hour incubation, CYP1A2 mRNA was quantified using real‐time quantitative reverse transcription polymerase chain reaction.

Following a 48‐hour incubation period, an enzymatic assay assessed phenacetin O‐deethylase (a CYP1A2 substrate) activity by measuring the CYP1A2‐dependent metabolite acetaminophen in supernatants. Acetaminophen was quantified using liquid chromatography coupled with tandem mass spectrometry (LC‐MS/MS).

For enzyme activity measurement, the criteria for data acceptance were considered met if the activity was higher than the limit of quantification of the method and if a successful induction at least equal to 2 times the control activity was obtained for all reference enzyme inducers. GLPG1205 was considered an enzyme inducer if the increase in enzyme activity was ≥40% of the response to the positive control. For the mRNA measurement, the in vitro study was considered positive for enzyme induction if incubations with GLPG1205 resulted in a more than 100% (ie, 2‐fold) increase in mRNA compared with the vehicle control, and the increase was concentration dependent.^14^ A concentration‐dependent increase in mRNA of <100% was considered a negative finding when the increase in mRNA was less than 20% of the response of the positive control.

#### In Vitro Assessment of CYP2C9, CYP2C19, and CYP1A2 Inhibition

Solubility experiments were performed prior to incubation of GLPG1205 with cells.

Inhibition of the biotransformation of CYP‐specific substrates by GLPG1205 was studied using human liver microsomes. Incubation was performed at 37°C with the following conditions: n = 3 for all assays; 0.1 M phosphate buffer (pH 7.4), the cofactor nicotinamide adenine dinucleotide phosphate (NADPH) incubated at 2 mM, the CYP‐specific substrate incubated at 1 nonsaturating concentration, and GLPG1205. Additional incubation was performed with (1) a 30‐minute preincubation step of GLPG1205 with microsomes before the addition of the cofactor NADPH and CYP substrate; and (2) a 30‐minute preincubation step of GLPG1205 with microsomes and the cofactor NADPH, before the addition of the CYP substrate. GLPG1205 was tested at 6 concentrations: 0.5, 1, 5, 10, 50, and 100 μM (0.19, 0.38, 1.89, 3.78, 18.9, and 37.8 μg/mL, respectively), for each condition. The CYP‐specific substrates were incubated per the above conditions at the following concentrations: tolbutamide, 200 μM, CYP2C9 substrate; S‐mephenytoin, 60 μM, CYP2C19 substrate; phenacetin, 40 μM, CYP1A2 substrate. Reference inhibitors were used to validate the inhibition screening assays for each enzyme and were incubated in parallel: sulfaphenazole (5 μM, CYP2C9 inhibitor); tranylcypromine (40 μM, CYP2C19 inhibitor); and furafylline (10 μM, CYP1A2 inhibitor).

The test concentrations were chosen based on the limit of solubility of GLPG1205 in the experimental conditions and were selected to be in close proximity to the maximum observed plasma concentration (C_max_) determined at steady state in the first‐in‐human study following multiple doses of 100 mg once a day.^4^ Further details of the assays used for each CYP substrate are reported in the Supplemental Methods. Metabolites of the CYP‐specific substrates (acetaminophen for CYP1A2, hydroxytolbutamide for CYP2C9, hydroxymephenytoin for CYP2C19) were quantified using LC‐MS/MS.

Enzymatic activity in the presence of GLPG1205 was expressed as a percentage of the control activity obtained with the vehicle only (ie, without GLPG1205 or reference inhibitor). Decreases in activity of less than 20% were not considered clinically relevant.

### Drug‐Drug Interaction Study

#### Study Design

This was a phase 1 single‐center, randomized, double‐blind, placebo‐controlled crossover drug‐drug interaction study (ClinicalTrials.gov number NCT02623296). The objectives of the study were to evaluate the effect of multiple oral doses of GLPG1205 on the single‐dose PK of a cocktail of CYP450 substrates and to evaluate the safety and tolerability of multiple doses of GLPG1205 administered with or without a cocktail of CYP450 substrates in healthy male subjects. The cocktail of CYP450 substrates contained CYP2C9 (warfarin 10 mg in tablet form), CYP2C19 (omeprazole 20 mg in capsule form), and CYP1A2 (caffeine 100 mg as an oral solution) substrates.^13^ Covariates that could impact GLPG1205 disposition are currently not known; therefore, to minimize variability that covariates could generate on GLPG1205 and subsequently warfarin, omeprazole, and caffeine pharmacokinetics, only male subjects were included with this study.

Subjects received treatment in 1 of 2 sequences (AB or BA, n = 7 in each sequence). In treatment A, oral doses of GLPG1205 100 mg once a day were administered from day 1 to day 12. On day 13, GLPG1205 100 mg (capsule form) was coadministered with the CYP450 substrates following an overnight fast. In treatment B, oral doses of placebo (capsule form) once a day were administered from day 1 to day 12. On day 13 placebo was coadministered with the CYP450 substrates following an overnight fast. Dose administration on day 13 was followed by a 4‐hour fast. The 2 treatment sequences were separated by a washout period of at least 28 days.

The screening visit occurred from 21 days to 1 day prior to administration of the first study drug. A follow‐up visit occurred on day 20 of treatment period 2. Subjects were housed from the evening of day 12 until approximately 26 hours after dosing on day 13. The study was conducted at a single investigational site (SGS Clinical Pharmacology Unit, Antwerp, Belgium) and in accordance with the Declaration of Helsinki and Good Clinical Practice guidelines and was approved by an independent ethics committee at the site.

#### Study Participants

All subjects provided written informed consent prior to enrollment. Key inclusion criteria were male and aged between 18 and 50 years, inclusive; with a body mass index between 18 and 30 kg/m², inclusive; judged to be in good health and had not received any medications (except occasional acetaminophen at a maximum dose of 2 g/day and 10 g/week) in the 3 weeks prior to administration of the first study drug; and a nonsmoker with negative urine drug screen. Exclusion criteria can be found in Supplemental Table 1.

#### Randomization and Blinding

When subjects were confirmed to be eligible for the study, they were assigned an identification number. Allocation of each subject to a treatment sequence was described in a randomization list prepared by the SGS Life Science Services Secure Data Office. Subjects, clinical study staff, and the sponsor were blinded to treatment.

#### Pharmacokinetic Assessments

Pharmacokinetic assessments included analysis of GLPG1205, and the CYP450 substrates and their metabolites in plasma. In each treatment period, blood samples were collected by venipuncture in the arm predose on days 7, 9, 10, and 13 and on day 13 at 1, 2, 4, 6, 8, 12, 24 (day 14), 48 (day 15), 96 (day 17), and 144 (day 19) hours postdose for PK analysis. For treatment period 2, an additional blood sample was also taken at 168 hours (day 20; follow‐up visit) postdose. Samples collected on days 7, 9, and 10 were for GLPG1205 analysis only. Blood samples of 2 mL were collected for the determination of GLPG1205 in plasma, and of 9 mL for the determination of CYP450 substrates and their metabolites. After collection, blood samples were immediately chilled using an ice bath. Within 30 minutes of collection, samples were separated by centrifugation at 1500 *g* for 10 minutes at 4°C. For GLPG1205, ≥250 μL of plasma was transferred into 2.0‐mL polypropylene tubes and stored at –20°C or below immediately after centrifugation at the clinical center until shipment to analysis centers. For CYP450 substrates, 500 μL of plasma was transferred to 2.0‐mL polypropylene tubes. Plasma samples were stored at –20°C or below (omeprazole/5‐hydroxyomeprazole) or –70°C or below (S‐warfarin/(S)‐7‐hydroxywarfarin and caffeine/paraxanthine) immediately after centrifugation at the clinical center until shipment to analysis centers. All samples were shipped on dry ice to analysis centers. Repetitive freeze‐thaw cycles were avoided to maintain the integrity of samples. Plasma concentrations were measured using LC‐MS/MS; further details can be found in the Supplemental Methods. Pharmacokinetic calculations were performed by SGS LSS using Phoenix WinNonLin 6.2 or higher (Pharsight Corporation, Palo Alto, California). The PK parameters determined for GLPG1205, S‐warfarin/(S)‐7‐hydroxywarfarin, omeprazole/5‐hydroxyomeprazole, and caffeine/paraxanthine included C_max_, t_max_, area under the plasma concentration‐time curve (AUC) from time zero until the time corresponding to the last observed quantifiable concentration (AUC_0–t_), AUC from time zero to infinity (AUC_0‐inf_), apparent terminal half‐life (t_1/2,λz_), and metabolite‐over‐parent ratio (R). Values below the quantification limit were imputed by 0 for the calculation of descriptive statistics except for geometric mean and geometric coefficient of variation (CV), for which it was imputed by half the lower limit of quantification.

#### Safety Assessments

The safety assessment was based on adverse events (AEs), vital signs, 12‐lead electrocardiogram (ECG), physical examinations, and safety laboratory tests. AEs were monitored for the duration of the study. All safety assessments were undertaken at the screening visit, on days 13 and 14, and at the follow‐up visit. In addition, safety assessments were undertaken on day 1 (vital signs, 12‐lead ECG, and clinical laboratory tests) and day 7 (physical examination, vital signs, and clinical laboratory tests).

#### Statistical Analysis

This is a pilot phase 1 study involving 14 subjects, and strict statistical criteria were not used to determine the sample size. The number of subjects included in this study gave reasonable precision around the estimates derived for the safety and PK evaluation. The PK population comprised all subjects who were exposed to GLPG1205/placebo and who had available and evaluable data. The safety population comprised all randomized subjects who received at least 1 dose of study drug.

An analysis of variance (ANOVA) with treatment, treatment period, and sequence as fixed effects and subject as a random effect was performed on natural log‐transformed PK parameters (R, C_max_, AUC_0‐inf_, AUC_0‐t_, and t_1/2,λz_) of CYP450 substrates and their metabolites on day 13. The treatment effect for t_max_ was assessed using the nonparametric Wilcoxon's signed rank test. Point estimates were calculated as the geometric mean of the individual ratios for each parameter for treatment A (GLPG1205 + CYP450 substrates) relative to treatment B (placebo + CYP450 substrates) and expressed as fold difference. The 90% confidence interval of the point estimate was calculated using the mean square error of the ANOVA. For GLPG1205, time to reach steady state was assessed by visual inspection of the trough plasma concentrations on days 7, 9, 10, and 13 (predose and 24 hours postdose).

## Results

### In Vitro Studies

#### In Vitro Assessment of CYP1A2 Induction

The limit of solubility of GLPG1205 in culture medium for incubations was 100 μM with a final DMSO percentage equal to 0.4%. According to morphological observations and neutral red uptake, GLPG1205 was found not cytotoxic over the range of 0‐50 μM and cytotoxic at 100 μM.

In the presence of 50 μM omeprazole (positive control), phenacetin O‐deethylase activity was increased by a factor of 53.9, 10.7, and 35.2 in the 3 hepatocyte batches (Supplemental Table 2). Therefore, the experiment met the validation criteria. All increases in CYP1A2 activity following incubation with GLPG1205 were lower than 40% of the positive control (omeprazole) and did not exceed 2‐fold compared with the negative control (0.4% DMSO; Supplemental Table 2).

In the presence of 50 μM omeprazole (positive control), CYP1A2 mRNA was increased by a factor of 341, 403, and 85.9 in the 3 hepatocyte batches (Supplemental Table 3). In the presence of GLPG1205 (0.1‐50 μM), 1 hepatocyte batch (HEP220794) showed no significant increase in CYP1A2 mRNA, with a maximum 1.60‐fold induction at 5 μM compared with vehicle control (Supplemental Table 3). In the other 2 hepatocyte batches, an increase in CYP1A2 mRNA expression (greater than 100% when compared with the vehicle control) was observed at the low concentrations, with a maximum 6‐fold induction at 1 μM GLPG1205 and lower fold induction observed at 5 to 50 μM GLPG1205 (Supplemental Table 3).

## In Vitro Assessment of CYP2C9, CYP2C19, and CYP1A2 Inhibition

All 3 experiments met the validation criteria with the percentage of inhibition obtained for the reference inhibitor was equal or greater than 50%.

Results demonstrated a moderate and reversible inhibition of CYP2C9 and CYP2C19 by GLP1205 50‐100 μM (Supplemental Figures 2 and 3). Inhibition of CYP2C9 ranged between 49.0% and 56.9% in the presence of GLPG1205 50‐100 μM without preincubation with similar percentages observed following a 30‐minute preincubation step with and without NADPH (Supplemental Figure 2). Similarly, CYP2C19 inhibition ranged between 44.2% and 49.3% in the presence of GLPG1205 50‐100 μM without preincubation, with similar percentages observed in both preincubation step conditions (with/without NADPH; Supplemental Figure 3). Weak in vitro inhibition of CYP1A2 was observed with GLPG1205 50‐100 μM. CYP1A2 inhibition ranged between 20.4% and 38.0% in the presence of GLPG1205 50‐100 μM, with 52.9% inhibition observed with GLPG1205 100 μM following a 30‐minute preincubation with NADPH (Supplemental Figure 4). The IC_50_ for inhibition of CYP2C9, CYP2C19, and CYP1A2 was estimated to be approximately 50 μM, ≥50 μM, and ≥100 μM, respectively.

### Drug‐Drug Interaction Study

#### Study Disposition and Demographics

**Figure 1:**
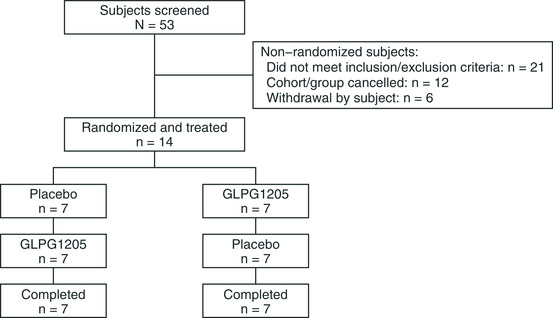
Flow chart to show details of subjects screened, randomized, and allocated to each treatment arm in the DDI study. DDI, drug‐drug interaction.

Fourteen healthy male subjects (median age, 41.5 years) were randomized to 1 of 2 treatment sequences (Table 1 and Figure 1). All randomized subjects completed the study. The study was conducted from September 24, 2015, until January 27, 2016.

**Table 1:** Demographics of Subjects in the DDI Study at Baseline

	All Subjects (n = 14)
Age, years	
Mean (SE)	40.6 (1.97)
Median (range)	41.5 (28‐49)
Weight, kg	
Mean (SE)	79.6 (2.79)
Median (range)	79.8 (67.7‐101.0)
BMI, kg/m^2^	
Mean (SE)	24.8 (0.70)
Median (range)	24.5 (20‐29)
Race, n (%)	
White	13 (92.9)
Unknown	1 (7.1)
Sex, n (%)	
Male	14 (100)

BMI, body mass index; SE, standard error.

#### PK Profile: GLPG1205

The PK parameters for GLPG1205 following 12 days of administration of GLPG1205 100 mg once a day are shown in Table 2. T_max_ was reached at 2 hours, with an apparent mean half‐life of 74.1 hours (Table 2). There were no statistically significant differences in the trough GLPG1205 plasma concentrations from day 9 to day 13, indicating steady state was reached by day 9 (ie, after 8 dosing days). In‐depth results on GLPG1205 PK parameters have previously been reported.^4^

**Table 2:** PK Parameters on Day 13, Following Once‐a‐Day Dosing of GLPG1205 100 mg for 12 Days

	GLPG1205 100 mg Once a Day Day 13 (n = 14)
C_max_ (μg/mL)	5.01 (23.1)
t_max_ (h)	2.0 (1.0‐8.0)
AUC_0‐t_ (μg·h/mL)	395 (36.8)
AUC_0‐inf_ (μg·h/mL)	373 (62.2)^n = 5^
t_1/2, λz_ (h)	74.1 (33.8)

AUC, area under the curve; AUC_0‐t_, from time zero until time corresponding to last observed quantifiable concentration; AUC_0‐inf_, from time zero to infinity; C_max_, plasma concentration; CV, coefficient of variation; PK, pharmacokinetic; t_1/2, λz_, apparent terminal half‐life; t_max_, time occurrence of C_max_.

Values are arithmetic mean (CV%), except for t_max_, which is: median (min‐max).

#### PK Profiles: (S)‐Warfarin and Its Metabolite (S)‐7‐Hydroxywarfarin (CYP2C9)

**Figure 2:**
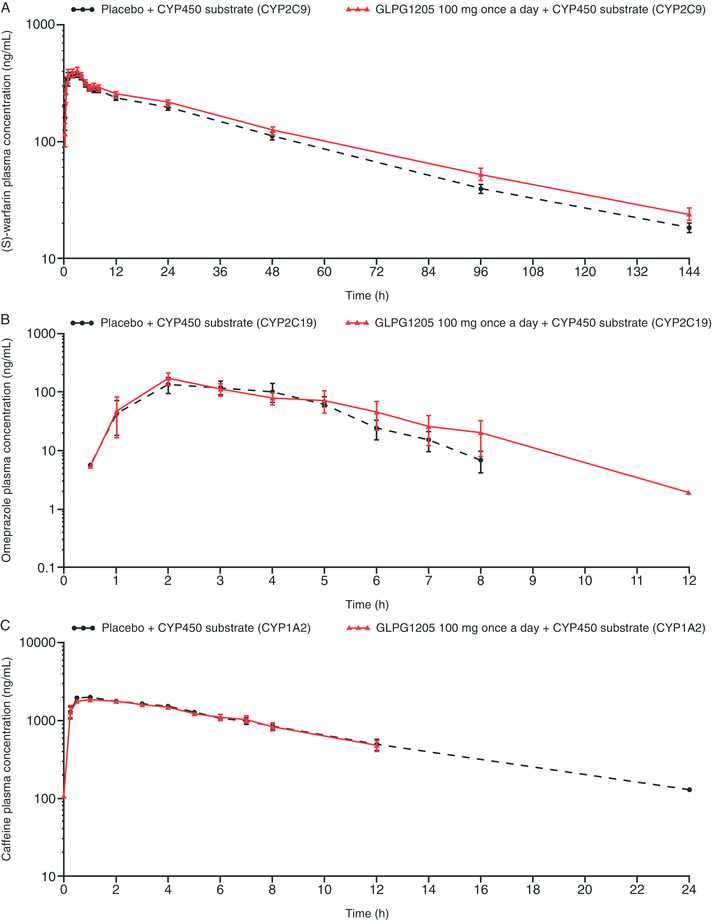
Effect of concomitant administration of GLPG1205 on plasma concentration‐versus‐time profiles of (A) (S)‐warfarin, (B) omeprazole, and (C) caffeine (mean ± standard error).

The plasma concentration‐versus‐time profiles were similar when (S)‐warfarin was administered with GLPG1205 or placebo (Figure 2A). The exposure of (S)‐warfarin was not affected when coadministered with GLPG1205 versus placebo, as the geometric mean ratio (90% confidence interval [CI]) for C_max_, AUC_0‐t_, AUC_0‐inf_, and t_1/2, λz_ were all within the 0.80‐1.25 fold difference range (Table 3A). The elimination phases were similar in both treatment groups, with (S)‐warfarin concentrations still quantifiable at the 144‐hour postdose measurement in all subjects (Figure 2A). The PK profile of (S)‐7‐hydroxywarfarin was not affected by GLPG1205 compared with placebo (Table 3B). (S)‐7‐hydroxywarfarin plasma concentrations were still quantifiable at 144 hours postdose in 12 of 14 subjects in each treatment group. The metabolite:parent ratios for (S)‐7‐hydroxywarfarin:(S)‐warfarin were similar when placebo or GLPG1205 was coadministered (0.156 and 0.158, respectively; Table 3B).

**Table 3:** PK Parameters for (A) (S)‐Warfarin and (B) (S)‐7‐Hydroxywarfarin

A			
	Placebo + Warfarin CYP2C9 Substrate, n = 14	GLPG1205 100 mg Once a Day + Warfarin CYP2C9 Substrate, n = 14	Comparison^a^ PE (90% CI) or *P*
C_max_ (μg/mL)	0.453 (21.3)	0.481 (19.8)	1.07 (1.00‐1.14)
t_max_ (h)	2.0 (0.5‐4.0)	2.0 (0.5‐4.0)	*P* = .5967
AUC_0‐t_ (μg·h/mL)	14.2 (20.0)	16.2 (22.4)	1.14 (1.07‐1.20)
AUC_0‐inf_ (μg·h/mL)	15.1 (21.1)	17.5 (24.9)	1.15 (1.08‐1.22)
t_1/2, λz_ (h)	33.9 (12.5)	36.5 (17.2)	1.07 (1.02‐1.12)
			
B			

	Placebo + Warfarin CYP2C9 Substrate, n = 14	GLPG1205 100 mg Once a Day + Warfarin CYP2C9 Substrate, n = 14	Comparison PE (90% CI) or *P*
C_max_ (μg/mL)	0.0347 (27.5)	0.0401 (27.8)	1.16 (1.01‐1.33)
t_max_ (h)	24.0 (24.0‐48.0)	24.0 (24.0‐48.0)	*P* = 1.000
AUC_0‐t_ (μg·h/mL)	2.25 (28.4)	2.62 (33.6)	1.15 (1.00‐1.32)
AUC_0‐inf_ (μg·h/mL)	2.57 (20.4)^n = 4^	2.95 (23.6)^n = 4^	—
t_1/2, λz_ (h)	32.2 (23.5)^n = 4^	46.9 (16.4)^n = 5^	—
R_AUC_0‐t_	0.156 (26.0)	0.158 (25.1)	1.01 (0.87‐1.19)

ANOVA, analysis of variance; AUC, area under the curve; AUC_0‐t_, from time zero until time corresponding to last observed quantifiable concentration; AUC_0‐inf_, from time zero to infinity; CI, confidence interval; C_max_, plasma concentration; CV, coefficient of variation; PE, point estimate; PK, pharmacokinetics; R, metabolite‐over‐parent ratio; t_1/2, λz_, apparent terminal half‐life; t_max_, time occurrence of C_max._

Values are arithmetic mean (CV%), except for t_max_, which is: median (min‐max).

^a^ PE and 90% CI of the least‐squares geometric means ratio of GLPG1205 relative to placebo (ANOVA); *P* value from Wilcoxon's signed rank test for t_max_.

#### PK Profiles: Omeprazole and Its Metabolite 5‐Hydroxyomeprazole (CYP2C19)

The maximal mean plasma concentrations of omeprazole was reached rapidly in both treatment groups, although there was some deviation in the elimination rates (Figure 2B). The exposure (AUC_0–t_, AUC_0–inf_, and C_max_) of omeprazole when coadministered with GLPG1205 was increased 1.28‐ to 1.36‐fold versus placebo, with an upper limit of the 90% CI of 1.49‐1.65 (Table 4A). Between‐subject variability in omeprazole C_max_ and AUC was high in both treatment groups (CV% between 69.3% and 83.9% with placebo and between 54.4% and 67.1% with GLPG1205; Table 4A). Omeprazole plasma concentrations were still quantifiable 12 hours postdose for 5 of 14 subjects in the placebo group and 3 of 14 subjects in the GLPG1205 group. The results indicate coadministration of either placebo or GLPG1205 had no effect on the clearance of 5‐hydroxyomeprazole (Table 4B). The 5‐hydroxyomeprazole plasma concentrations were still quantifiable 12 hours postdose for 7 of 14 subjects in the placebo group and 8 of 14 subjects in the GLPG1205 group.

**Table 4:** PK Parameters for (A) Omeprazole and (B) 5‐Hydroxyomeprazole

A			
	Placebo + Omeprazole CYP2C19 Substrate, n = 14	GLPG1205 100 mg Once a Day + Omeprazole CYP2C19 Substrate, n = 14	Comparison^a^ PE (90% CI) or *P*
C_max_ (μg/mL)	0.219 (69.3)	0.270 (54.4)	1.30 (1.03‐1.65)
t_max_ (h)	3.0 (2.0‐5.0)	2.0 (1.0‐5.0)	*P* = .8281
AUC_0‐t_ (μg·h/mL)	0.486 (83.9)	0.563 (63.5)	1.28 (1.10‐1.49)
AUC_0‐inf_ (μg·h/mL)	0.520 (78.5)^n = 13^	0.548 (67.1)^n = 13^	1.36 (1.18‐1.58)
t_1/2, λz_ (h)	0.815 (30.7)^n = 13^	0.825 (24.7)^n = 13^	1.09 (0.97‐1.22)
			
B			

	Placebo + Omeprazole CYP2C19 Substrate, n = 14	GLPG1205 100 mg Once a Day + Omeprazole CYP2C19 Substrate, n = 14	Comparison^a^ PE (90% CI) or *P*
C_max_ (μg/mL)	0.149 (26.7)	0.162 (38.0)	1.05 (0.88‐1.25)
t_max_ (h)	3.0 (2.0‐5.0)	2.5 (2.0‐6.0)	*P* = .9922
AUC_0‐t_ (μg·h/mL)	0.383 (21.6)	0.414 (22.7)	1.08 (0.96‐1.21)
AUC_0‐inf_ (μg·h/mL)	0.388 (20.9)	0.422 (21.7)	1.08 (0.98‐1.20)
t_1/2, λz_ (h)	1.18 (22.1)	1.37 (23.7)	1.16 (1.04‐1.30)
R_AUC_0‐t_	1.06 (76.7)	0.889 (68.4)	0.84 (0.79‐0.89)
R_AUC_0‐inf_	0.983 (69.7)^n = 13^	0.958 (64.2)^n = 13^	0.83 (0.77‐0.89)

ANOVA, analysis of variance; AUC, area under the curve; AUC_0‐t_, from time zero until time corresponding to the last observed quantifiable concentration; AUC_0‐inf_, from time zero to infinity; CI, confidence interval; C_max_, plasma concentration; CV, coefficient of variation; PE, point estimate; PK, pharmacokinetics; R, metabolite‐over‐parent ratio; t_1/2, λz_, apparent terminal half‐life; t_max_, time occurrence of C_max._

Values are arithmetic mean (CV%), except for t_max_, which is median (min‐max), and for R_AUC_0‐t_ and R_AUC_0‐inf_, which are geometric mean (geometric CV%).

^a^ PE and 90% CI of the least‐squares geometric means ratio of GLPG1205 relative to placebo (ANOVA); *P* value from Wilcoxon's signed rank test for t_max_.

#### PK Profiles: Caffeine and Its Metabolite Paraxanthine (CYP1A2)

Predose caffeine plasma concentrations were above the lower limit of quantification (>100 ng/mL) and at a significant level (>5% C_max_) for 2 subjects in each treatment group. Caffeine was also quantifiable in all subjects at the first sampling point (15 minutes postdose). The maximal mean plasma concentration for caffeine was rapidly reached and similar in both treatment groups (Figure 2C). The exposure of caffeine was similar in the presence of placebo or GLPG1205 (Table 5A). For caffeine, the elimination phase was monophasic and superimposed in both treatment groups (Figure 2C). Caffeine plasma concentrations were still quantifiable 24 hours postdose for 6 of 14 subjects in both treatment groups. The PK profile of paraxanthine was similar in the presence of placebo or GLPG1205; overall PK parameters were similar between treatment groups, and the 90% CIs were close to or within the 0.80‐ to 1.25‐fold range (Table 5B). Paraxanthine plasma concentrations were still quantifiable 24 hours postdose for 10 and 7 of 14 subjects in the placebo and GLPG1205 groups, respectively. The metabolite:parent ratio for paraxanthine:caffeine was similar following coadministration with placebo or GLPG1205 (Table 5B).

**Table 5:** PK Parameters for (A) Caffeine and (B) Paraxanthine

A			
	Placebo + Caffeine CYP1A2 Substrate, n = 14	GLPG1205 100 mg Once a Day + Caffeine CYP1A2 Substrate, n = 14	Comparison^a^ PE (90% CI) or *P*
C_max_ (μg/mL)	2.15 (12.9)	2.16 (25.1)	0.99 (0.88‐1.11)
t_max_ (h)	1.0 (0.3‐3.0)	0.9 (0.3‐4.0)	*P* = .4492
AUC_0‐t_ (μg·h/mL)	16.3 (38.7)	15.7 (43.5)	0.96 (0.86‐1.06)
AUC_0‐inf_ (μg·h/mL)	18.7 (39.6)^n = 12^	17.4 (42.5)	0.93 (0.81‐1.07)
t_1/2, λz_ (h)	5.73 (45.9) ^n = 13^	4.90 (32.2)	0.92 (0.83‐1.01)
			
B			

	Placebo + Caffeine CYP1A2 Substrate, n = 14	GLPG1205 100 mg Once a Day + Caffeine CYP1A2 Substrate, n = 14	Comparison^a^ PE (90% CI) or *P*
C_max_ (μg/mL)	0.577 (14.8)	0.634 (22.8)	1.08 (0.97‐1.21)
t_max_ (h)	7.0 (3.0‐12.0)	7.0 (0.3‐12.0)	*P* = .3564
AUC_0‐t_ (μg·h/mL)	8.43 (24.5)	8.60 (48.2)	0.96 (0.79‐1.16)
R_AUC_0‐t_	0.539 (30.5)	0.538 (27.7)	1.00 (0.87‐1.15)

ANOVA, analysis of variance; AUC, area under the curve; AUC_0‐t_, from time zero until time corresponding to the last observed quantifiable concentration; AUC_0‐inf_, from time zero to infinity; CI, confidence interval; C_max_, plasma concentration; CV, coefficient of variation; PE, point estimate; PK, pharmacokinetics; R, metabolite over parent ratio; t_1/2, λz_, apparent terminal half‐life; t_max_, time occurrence of C_max._

Values are arithmetic mean (CV%), except for t_max_, which is: median (min‐max), and for R AUC_0‐t_, which is geometric mean (geometric CV%).

^a^ PE and 90% CI of the least‐squares geometric means ratio of GLPG1205 relative to placebo (ANOVA); *P* value from Wilcoxon's signed rank test for t_max_.

#### Safety and Tolerability Profile

With placebo alone, 6 subjects reported a treatment‐emergent AE (TEAE), and with GLPG1205 alone, 9 subjects reported a TEAE (Table 6A). The most commonly reported TEAEs with GLPG1205 or placebo alone were headache (GLPG1205, n = 5; placebo, n = 3), gastroenteritis (GLPG1205, n = 1; placebo, n = 1), and disturbance in attention (GLPG1205, n = 2). All cases of headache and disturbance in attention were considered treatment related. In both GLPG1205 plus CYP450 and placebo plus CYP450 substrate treatment groups, 3 subjects reported a TEAE (Table 6B). The most commonly reported TEAE during CYP450 substrate administration was headache (GLPG1205 plus CYP450 substrates, n = 1; placebo plus CYP450 substrates, n = 1). All TEAEs reported were mild in intensity except for moderate headache (placebo alone, n = 1), moderate presyncope (GLPG1205 alone, n = 1), and moderate oropharyngeal pain (GLPG1205 plus CYP450 substrates, n = 1). No clinically significant trends or abnormalities were detected with laboratory safety tests, ECG, vital signs, or physical examination. There were no deaths or serious AEs during the study.

**Table 6:** Incidence of Adverse Events With (A) Placebo or GLPG1205 Alone and (B) Placebo or GLPG1205 Plus CYP450 Substrates

A		
System Organ Class Preferred Term, n (%)	Placebo, n = 14	GLPG1205 100 mg Once a Day, n = 14
Any TEAE	6 (42.9)	9 (64.3)
Nervous system disorders	3 (21.4)	8 (57.1)
Headache	3 (21.4)	5 (35.7)
Disturbance in attention	0	2 (14.3)
Paresthesia	0	1 (7.1)
Presyncope	0	1 (7.1)
General disorders and general administration‐site conditions	0	1 (7.1)
Fatigue	0	1 (7.1)
Musculoskeletal and connective tissue disorders	1 (7.1)	1 (7.1)
Myalgia	0	1 (7.1)
Back pain	1 (7.1)	0
Eye disorders	1 (7.1)	1 (7.1)
Lacrimation increased	0	1 (7.1)
Vision blurred	1 (7.1)	0
Gastrointestinal disorders	0	1 (7.1)
Flatulence	0	1 (7.1)
Infections and infestations	2 (14.3)	1 (7.1)
Gastroenteritis	1 (7.1)	1 (7.1)
Nasopharyngitis	1 (7.1)	0
Skin and subcutaneous tissue disorders	2 (14.3)	0
Hyperhidrosis	1 (7.1)	0
Rash	1 (7.1)	0
Respiratory, thoracic, and mediastinal disorders	0	1 (7.1)
Oropharyngeal pain	0	1 (7.1)
Vascular disorders	0	1 (7.1)
Flushing	0	1 (7.1)
		
B		

System Organ Class Preferred Term, n (%)	Placebo + CYP450 Substrates, n = 14	GLPG1205 100 mg Once a Day + CYP450 Substrates, n = 14
Any TEAE	3 (21.4)	3 (21.4)
Nervous system disorders	1 (7.1)	1 (7.1)
Headache	1 (7.1)	1 (7.1)
General disorders and general administration‐site conditions	2 (14.3)	0
Influenza‐like illness	1 (7.1)	0
Pain	1 (7.1)	0
Musculoskeletal and connective tissue disorders	0	1 (7.1)
Myalgia	0	1 (7.1)
Gastrointestinal disorders	1 (7.1)	0
Nausea	1 (7.1)	0
Respiratory, thoracic, and mediastinal disorders	0	1 (7.1)
Oropharyngeal pain	0	1 (7.1)

TEAE, treatment‐emergent adverse event.

## Discussion

As part of the development of GLPG1205 for the treatment of IPF, we investigated the potential of GLPG1205 to interact with other drugs. As GLPG1205 may be administered with pirfenidone in future clinical trials, a special focus was made on the CYP450 enzymes involved in the metabolism of pirfenidone (CYP1A2, CYP2C9, and CYP2C19).^8^, ^9^

Results of the in vitro studies indicated there was weak inhibition of CYP1A2 (with no interpretation about reversibility) and moderate but reversible inhibition of CYP2C9 and CYP2C19 by GLPG1205. The estimated IC_50_ values for inhibition of CYP2C9/2C19 (≥50 μM, equivalent to 18.9 μg/mL) and CYP1A2 (≥100 μM, equivalent to 37.8 μg/mL) observed in the current study are approximately 5‐ to 10‐fold greater than the C_max_ of 3.91 μg/mL reached following multiple doses of GLPG1205 100 mg once a day (the maximum tolerated dose) in the first‐in‐human study characterizing PK properties of GLPG1205.^4^ The in vitro assessment of CYP1A2 induction revealed that GLPG1205 increased CYP1A2 mRNA expression more than 100% compared with the vehicle control. On the basis of these results and given that the cumulative impact of weak/moderate effects on multiple CYP450 enzymes is difficult to predict, it was necessary to ensure GLPG1205 did not interfere with the pirfenidone metabolism pathways by conducting a clinical study.

The PK properties of GLPG1205 100 mg once a day observed in the current study were similar to those previously reported in a first‐in‐human study, with rapid absorption, a long apparent terminal elimination half‐life, and similar steady‐state plasma concentrations.^4^ Comparison of the results from both studies demonstrated that subjects in the current drug‐drug interaction study were adequately exposed to GLPG1205 under steady‐state conditions at the projected (or maximum tolerated) 100‐mg once‐a‐day dose.

The coadministration of CYP450 substrates on day 13, following 12 days of GLPG1205 100‐mg once‐a‐day dosing alone, resulted in no significant effect on the exposure of the CYP2C9 (warfarin), CYP2C19 (omeprazole), and CYP1A2 (caffeine) substrates or the PK profiles of their metabolites. Despite an increase in omeprazole exposure, there was no increase in the elimination rate of omeprazole. The elimination rate of 5‐hydroxyomeprazole was 1.16‐fold higher with GLPG1205 versus placebo, but this was not considered clinically relevant because of the high between‐subject variability of omeprazole exposure (54%‐84%), and there was only a slight impact on the parent:metabolite ratio for omeprazole:5‐hydroxyomeprazole. Overall, this study confirmed that GLPG1205 does not interact with CYP2C9, CYP2C19, or CYP1A2 in humans to a clinically relevant extent and therefore provides reassurance that GLPG1205 can be administered with pirfenidone in future clinical trials.

Multiple oral doses of GLPG1205 100 mg once a day administered with or without CYP450 substrates had safety and tolerability profiles comparable to the first‐in‐human study,^4^ with no new safety signals observed in the current study.

Some limitations must be acknowledged. There was a small sample size and homogeneity of subjects in the clinical study; however, these were appropriate for a phase 1 study.

## Conclusions

GLPG1205 does not interact with CYP2C9, CYP2C19, and CYP1A2 in humans to a clinically relevant extent, and therefore, GLPG1205 may be administered concomitantly with drugs that are metabolized by these enzymes.

## Conflicts of Interest

T.V.K. and E.H. are employees of Galapagos. J.D. and L.A. are former employees of Galapagos. No COIs were reported by spouses/partners of the authors.

## Funding

The study and writing/editorial support were funded by Galapagos, Mechelen, Belgium.

## Author Contributions

All authors provided substantial contributions to the design or the acquisition, analysis, or interpretation of data. Authors critically reviewed the article for important intellectual content. All authors read and approved the final version of the article.

## Supporting information

Supplemental Information

## Data Availability

Data will not be shared.
