# Supplementary material for: Effect of GLPG1205, a GPR84 Modulator, on CYP2C9, CYP2C19, and CYP1A2 Enzymes: In Vitro and Phase 1 Studies
Source: Clin Pharmacol Drug Dev. 2021 May 6;10(9):1007–17. doi: 10.1002/cpdd.956 (PMC8453848; doi:10.1002/cpdd.956)

**Supplementary Methods**

***In Vitro Assessment of CYP1A2 Induction***

**Test of Solubility** Prior to incubation of GLPG1205 with cells, a preliminary test of solubility was performed. Various stock solutions of GLPG1205 in dimethylsulfoxide (DMSO) were prepared. Different solutions were prepared by diluting the stock solutions in culture medium for incubation to obtain 100, 200, 250, 400 and 500 µM with different final percentages of solvent (maximum 0.5% DMSO, volume/volume [v/v]). The solubility was assessed by macroscopic observation just after solubilization, and by microscopic observation just after solubilization and after 24 hours of incubation at 37°C.

**Cytotoxicity experiment** GLPG1205 was incubated with human hepatocytes for 48 hours (± 4 hours), at 6 concentrations: 0.5, 1, 5, 10, 50, 100 µM (0.19, 0.38, 1.89, 3.78, 18.9 and 37.8 µg/mL, respectively). All incubations contained 0.4% DMSO. The test concentrations for the cytotoxicity experiment were chosen based on: the limit of solubility of GLPG1205 in experimental conditions (see results section); and proximity to the plasma concentration (C_max_) value found in humans at steady-state in the first-in-human study of GLPG1205 (ie, 3.91 µg/mL following multiple doses of 100 mg once a day [QD]).^1^ The incubations were performed with cell monolayers in a 96-well plate with a volume of 100 µL per well.^2^ The culture medium for incubations (CMI) was William’s E medium with hepatocytes induction supplements. Cells were cultured at 37°C in a humidified carbon dioxide/air (5%/95%) atmosphere.

Control incubations performed in parallel included: cultures without test compound, incubated for 48 hours (± 4 hours) with CMI containing 0.4% DMSO (v/v); and test compound incubated without cells for 48 hours (± 4 hours; blank value). Culture media were renewed daily in all wells and cell morphology was monitored (by light microscopy before incubation, and at 24 and 48 hours of incubation) to detect any signs of cytotoxicity.

At the end of the incubation period, cytotoxicity was assessed using 2 end-points: morphological observations and measurement by spectrophotometry of neutral red uptake.

To assess neutral red uptake, at the end of the incubation period with the test compound, cells were incubated for approximately 3 hours with CMI containing neutral red dye. After microscopic assessment of dye uptake, the dye-medium was removed and the cells were washed with formaldehyde-CaCl_2_ to remove unincorporated dye and promote adhesion of the cells. Removal of the formaldehyde solution and addition of acetic acid-ethanol solution (50/50, v/v) resulted in the extraction of the neutral red dye into the solution. The optical density was then measured with a spectrophotometer at λ = 540 nm.

**Incubation for CYP1A2 Induction Experiment** The incubations for enzyme activity measurements were performed with cell monolayers in 96-well plates and the incubations for mRNA measurements were performed with cell monolayers in 24-well plates.^3^

GLPG1205 was incubated with monolayer culture of human fresh hepatocytes from 3 individual donors for 24 or 48 hours, for messenger ribonucleic acid (mRNA) analysis and enzyme activity, respectively, at 6 different concentrations (n = 3): 0.1, 0.5, 1, 5, 10 and 50 µM (0.038, 0.19, 0.38, 1.89, 3.78 and 18.9 µg/mL, respectively). Incubations contained 0.4% DMSO based on the limit of solubility test for GLPG1205. Incubation with 50 µM omeprazole containing 0.1% DMSO was used as a positive control. Incubations with 0.1% and 0.4% DMSO alone were used as negative controls. The CMI was William’s E medium with hepatocytes induction additives. Cells were cultured at 37°C in a humidified carbon dioxide/air (5%/95%) atmosphere. Culture media were renewed daily in all wells.

**Enzymatic Assay** At the end of the 48-hour incubation period, CYP1A2 enzyme activity was measured in intact cells by incubating with phenacetin, a probe substrate. Cells were first washed with minimum essential medium (MEM) devoid of phenol red to remove remaining test compounds or reference inducers and were then incubated for 2 hours at 37°C with 200 µM phenacetin in MEM devoid of phenol red and containing 0.1% DMSO. Assessment of phenacetin O-deethylase activity in supernatants was carried out by measuring the CYP1A2-dependent metabolite acetaminophen. Cell protein content was also determined; protein content was measured according to the bicinchoninic acid method^4^ and calculated using a bovine serum albumin standard curve.

**mRNA Analysis** At the end of the 24-hour incubation period, the incubation medium was removed and the cell monolayers were washed twice with 500 µL phosphate buffered saline. The cells were lysed using 350 µL per well of cell lysis buffer and were immediately extracted on columns from the Macherey–Nagel^®^ kit. Total RNA and RNA quality were assessed by measuring 260/280 nm and 260/230 nm absorbance ratios. RNA concentrations were used to normalize the quantity of RNA used for the reverse transcriptase assay. Total mRNA was then reversed as complementary deoxyribonucleic acid (cDNA) using High capacity cDNA reverse transcription (RT) Kit with RNAse inhibitor.

Quantitative real-time reverse transcription polymerase chain reaction (qRT-PCR) was performed using SYBRGreen^®^ validated primers for CYP1A2.^3^ The housekeeping gene glyceraldehyde 3-phosphate dehydrogenase (GAPDH) was analyzed in parallel as the control gene. The qRT-PCR amplification and transcription was performed using Applied Biosystem 7900HT Fast Real-Time PCR System. A CYP1A2 mRNA standard was created by pooling cells induced by 50 µM omeprazole. The pool was given a nominal concentration of 1 and standard curves were prepared by serial dilution to determine the limits of cycle time linearity.

**Liquid Chromatography Coupled with Tandem Mass Spectrometry (LC-MS/MS) Analysis** Specimens were analyzed by LC-MS/MS, using an Applied Biosystems API3200 triple quadrupole mass spectrometer, with a Turbo-V ionization source. The mass spectrometer was operated in the Multiple-Reaction-Monitoring (MRM) mode in positive polarity. The 151.9 to 109.9 transition for acetaminophen was used for quantification.

A 2-fold dilution of the specimens in a solution of 1 µM D4-acetaminophen in water with 0.1% formic acid was performed and 20 µL were injected into the LC-MS/MS system. The mobile phase consisted of ammonium acetate 5 mM in water (A) and formic acid 0.3% in acetonitrile/methanol, 50/50, v/v. The following gradient was applied with a 1.5 mL/min flow rate and an oven temperature of 40°C: 98% A from 0 minutes; 70% A from 2.5 minutes; 1% A from 2.6 minutes; 1% A from 3.2 minutes; 98% A from 3.25 minutes; 98% A from 4.5 minutes. In this condition, the retention time of acetaminophen was approximately 2.33 minutes. Controls were performed by injecting calibration standards at the beginning and end of each run. Each calibration curve was obtained by linear regression of peak area of metabolite versus theoretical concentration (X) with a weighting factor of 1/X.

**Data Treatment** For the cytotoxicity experiment, the ratio of optical density of treated versus control cells was calculated and expressed as a percentage of viability, for every concentration of test compound.

For enzyme activity measurement, the criteria for data acceptance were considered met if the activity was higher than the limit of quantification of the method, and if a successful induction at least equal to 2 times the control activity was obtained for all reference enzyme inducers. The change in enzyme activity produced by the test compound was compared with the positive control as follows:^5^

$$\% of positive control=\frac{\left( activity of treated cells-activity of negative control \right) x 100}{(activity of postive control-activity of negative control)}$$

GLPG1205 was considered as an enzyme inducer if the increase in enzyme activity is ≥40% of the response to positive control.^5^

For mRNA measurement, the threshold cycle (C_Τ_) value and the fractional cycle number at which the fluorescence passes the fixed threshold were determined for each incubation. mRNA content for CYP1A2 was related the housekeeping gene (GAPDH) and an analysis of the results was then performed to determine potential differences from the control data. The fold induction (FI) was determined as:

$$\Delta C_{T}= C_{T \left( interest gene \right)}- C_{T (GADPH)}$$

$$FI=\frac{2^{-\Delta T(test\_compound)}}{2^{-\Delta T(negative\_control)}}$$

Maximal observed induction factor was compared with positive control inducer for each test compound concentration according to the following formula:

$$\%\left( positive control \right)=\frac{{(FI}_{test\_compound}- {FI}_{negative\_control})}{{(FI}_{positive\_control}- {FI}_{negative\_control})}x 100$$

The in vitro study was considered positive for enzyme induction if incubations with GLPG1205 resulted in a more than 100% (ie 2-fold) increase in mRNA as compared with the vehicle control, and the increase was concentration dependent. A concentration-dependent increase in mRNA of <100% was considered as a negative finding when the increase in mRNA was less than 20% of the response of the positive control.

***In Vitro Assessment of CYP1A2, CYP2C9 and CYP2C19 Inhibition***

**Test of Solubility** Prior to incubation of GLPG1205 with microsomes, a preliminary test of solubility was performed on a stock solution of GLPG1205 at 40 mM in different solvents (methanol, ethanol and DMSO). Macroscopic and microscopic observation was performed after solubilization. A test of solubility was also performed on the final solution of GLPG1205 at 400 and 300 µM (prepared by a 1/100 dilution of stock solutions at 40 and 30 mM, respectively, in phosphate buffer), and at 200, 150, and 100 µM (prepared by a 1/200 dilution of stock solutions at 40, 30 and 20 mM, respectively, in phosphate buffer). The solubility was assessed by macroscopic and microscopic observations immediately, and after 1 and 2 hours at room temperature.

**Preparation of Solutions for Incubation** Based on the solubility assay, a stock solution of 20 mM GLPG1205 in DMSO was prepared on each experimental day. Successive dilutions in DSMO of this stock solution were performed to prepare intermediate stock solutions 200 times more concentrated than the final solution. The stock solution and intermediate stock solution were directly diluted in the incubation medium (0.1 M phosphate buffer, pH 7.4) by a 1/200 dilution to reach the final concentrations of test compound with solvent at 0.5% v/v.

Reference inhibitors were used to validate the inhibition screening assays for each cytochrome. Sulfaphenzole (CYP2C9) contained DMSO 0.5% v/v and the compound at 5 µM, tranylcypromine (CYP2C19) contained methanol 0.5% v/v and the compound at 40 µM, furafylline (CYP1A2) contained DMSO 0.5% v/v and the compound at 10 µM.^6,7^

**Protocol for CYP Inhibition** of the biotransformation of CYP-specific substrates by GLPG1205 was studied using human liver microsomes.^7^ Incubations were performed at 37°C with the following conditions: n = 3 for all assays; 0.1 M phosphate buffer pH 7.4; the cofactor β-nicotinamide adenine dinucleotide phosphate (NADPH) incubated at 2 mM; the CYP-specific substrate incubated at one non-saturating concentration; and GLPG1205. Additional incubations were performed with: 1) a 30-minute preincubation step of GLPG1205 with microsomes before addition of the cofactor NADPH and CYP substrate; and 2) a 30-minute preincubation step of GLPG1205 with microsomes and the cofactor NADPH, before addition of the CYP substrate.

GLPG1205 was tested at 6 concentrations: 0.5, 1, 5, 10, 50 and 100 µM (0.19, 0.38, 1.89, 3.78, 18.9, 37.8 µg/mL, respectively). The test concentrations were chosen based on the limit of solubility of GLPG1205 in experimental conditions, and were selected to be in close proximity to the C_max_ value found at steady-state in the first-in-human study following multiple doses of 100 mg QD (ie, 3.91 µg/mL following multiple doses of 100 mg QD).^1^

The CYP-specific substrates were incubated at the following conditions: tolbutamide (200 µM; CYP2C9); S-mephenytoin (60 µM; CYP2C19); phenacetin (40 µM; CYP1A2).^8-10^ Reference inhibitors (as detailed in the previous section) were incubated in parallel. For all assays, the following controls were incubated in parallel: 1) with CYP substrate, without microsomes, with NADPH, without inhibitor (to check for substrate stability); 2) with CYP substrate, with microsomes, without NADPH, without inhibitor (to check for non-NADPH-dependent metabolism of substrate); and 3) without substrate, with microsomes, with NADPH, with test compound or reference inhibitor (to check for potential analytical interference).

**CYP2C9: Tolbutamide Methylhydroxylase Assay** The microsomal suspension (0.8 mg/mL^-1^) was incubated for 20 minutes in a final volume of 300 µL in the presence of GLPG1205 or sulfaphenazole 5 µM, and in the presence of tolbutamide 200 µM (substrate) and the cofactor NADPH 2 mM. At the end of the incubation period, the reaction was stopped with the addition of 0.15 M phosphoric acid (300 µL). After centrifugation at 3200 *g* for 15 minutes at approximately + 10°C, supernatants were collected.

A 2-fold dilution in D9-OH-tolbutamide at 1 µM in water with 0.1% formic acid was applied to the supernatants and then analysis was performed by LC-MS/MS with chromatographic separation. The mobile phase consisted of: 5 mM ammonium acetate and acetronitrile/methanol (50/50, v/v), with 0.3% (v/v) formic acid. The quantification of the metabolite, hydroxytolbutamide, was performed in MRM mode using the 287.1 to 89.2 transition.

**CYP2C19: S-mephenytoin 4’-hydroxylase Assay** The microsomal suspension (0.8 mg.mL^-1^) was incubated for 20 minutes in a final volume of 300 µL in the presence of GLPG1205 or tranylcypromine, and in the presence of S-mephenytoin 60 µM and the cofactor NADPH 2 mM. At the end of the incubation period, the reaction was stopped with the addition of 0.15 M phosphoric acid (300 µL); proteins were allowed to precipitate for a minimum of 1 hour at approximately 4°C. After centrifugation at 3200 g for 20 minutes at approximately + 4°C, supernatants were collected.

A two-fold dilution in D3-OH-mephenytoine at 1 µM in water with 0.1% formic acid was applied to the supernatants and then analysis was performed by LC-MS/MS with chromatographic separation. The mobile phase consisted of: 5 mM ammonium acetate and acetronitrile/methanol (50/50, v/v), with 0.3% (v/v) formic acid. The quantification of the metabolite, hydroxymephenytoin, was performed in MRM mode using the 235.1 to 150.2 transition.

**CYP1A2: Phenacetin O-deethylase Assay** The microsomal suspension (0.3 mg.mL^-1^) was incubated for 7 minutes in a final volume of 300 µL in the presence of GLPG1205 or furafylline, and in the presence of phenacetin 40 µM and the cofactor NADPH 2 mM. At the end of the incubation period, the reaction was stopped with the addition of 0.15 M phosphoric acid (300 µL); proteins were allowed to precipitate for a minimum of 1 hour at approximately 4°C. After centrifugation at 3200 g for 20 minutes at approximately + 4°C, supernatants were collected.

A 2-fold dilution in D4-acetaminophen at 1 µM in water with 0.1% formic acid was applied to the supernatants and then analysis was performed by LC-MS/MS with chromatographic separation. The mobile phase consisted of: 5 mM ammonium acetate and acetronitrile/methanol (50/50, v/v), with 0.3% (v/v) formic acid. The quantification of the metabolite, acetaminophen, was performed in MRM mode using the 152 to 110 transition.

**Data Treatment of Enzymatic Activities** The enzymatic activities in the presence of GLPG1205 were expressed as a percent of the control activities obtained with the vehicle only (ie, without GLPG1205 or reference inhibitor). Decreases in activity of less than 20% were not considered to be clinically relevant. The experiment was validated when the percentage of inhibition obtained for the reference inhibitor was ≥50%.

***Measurement of GLPG1205, (S)-warfarin, (S)-7-hydroxywarfarin, Omeprazole, 5-hydroxyomeprazole, Caffeine and Paraxanthine in Human Plasma***

**GLPG1205** The GLPG1205 concentrations in the plasma were determined by using a validated LC/MS-MS method. Prior to injection into the analytical system, isolation of GLPG1205 and its stable isotope labeled internal standard (IS) (G447434 – ^13^C labelled GLPG1205) from 20 µL of human Li-heparin plasma was performed by protein precipitation with 600 µL of methanol. The protein precipitated mixture was centrifuged at approximately 14 000 *g* for 15 minutes at room temperature. The clear supernatant was diluted with dilution solvent (water with 0.05% of trifluoroacetic acid) and then transferred into LC-MS plates. 5 µL of the reconstituted sample was injected into the chromatographic system. Chromatographic separation was performed on a Kinetex C18 column (50 x 3.0 mm, 2.6 µm from Phenomenex, Torrance, CA, USA) set at 40°C by using a Nexera high performance liquid chromatography (HPLC) system (Shimadzu, Kyoto, Japan) in isocratic elution mode. The aqueous mobile phase consisted of a mixture of acetonitrile:water (40:60, v/v) containing 0.05% of trifluoroacetic acid. A QTRAP6500 mass spectrometer (AB Sciex, Nieuwerkerk aan den Ijssel, The Netherlands) equipped with a TIS probe operated in the multiple reaction monitoring (MRM) in positive mode was used for quantification. The precursor‐to‐product ion pairs at the mass‐to‐charge ratio (m/z) were 379.1 to 279.1 and 382.1 to 282.1 for GLPG1205 and G447434, respectively. The calibration curves in plasma were linear over the range of 1–1000 ng/mL with 1/x^2^ as weighting factor. The limit of quantification of the assay in the plasma samples was set at 1 ng/mL.

**(S)-warfarin and Its Metabolite (S)-7-hydroxywarfarin** (S)-warfarin and its metabolite (S)-7-hydroxywarfarin concentrations in the plasma were determined by using a validated LC/MS-MS method. Prior to injection into the analytical system, isolation of (S)-warfarin, its metabolite and their respective deuterated IS (Warfarin-d5 and 7-hydroxywarfarin-d5) from 100 µL of human Li-heparin plasma was performed by protein precipitation with 500 µL of acetonitrile. The protein precipitated mixture was centrifuged at approximately 3500 rpm for 5 minutes at +5°C. The clear supernatant was diluted with a mixture of water/acetonitrile (60:40, v/v) with 0.1% of acetic acid and then transferred into LC-MS plates. 5 µL of the reconstituted sample was injected into the chromatographic system. Chromatographic separation was performed on a Chiral OD-RH column (150 x 2.1 mm, 5 µm from Chiral technologies/Daicel, Illkirch, France) set at 40°C by using an HLPC system (Shimadzu, Kyoto, Japan) in isocratic elution mode. The aqueous mobile phase consisted of a mixture of acetonitrile:water (50:50, v/v) containing 0.1% of acetic acid. An API4000 mass spectrometer (AB Sciex, Nieuwerkerk aan den Ijssel, The Netherlands) equipped with a TIS probe operated in the MRM in positive mode was used for quantification. The precursor‐to‐product ion pairs at the mass‐to‐charge ratio (m/z) were 309.1 to 163.2, 324.9 to 178.9 and 314.2 to 163.1, 330.1 to 179.1 for (S)-warfarin, its metabolite and for their respective IS. The calibration curves in plasma for both compounds were linear over the range of 2–2000 ng/mL with 1/x^2^ as weighting factor. The limit of quantification of the assay in the plasma samples was set at 2 ng/mL for (S)-warfarin and its metabolite.

**Omeprazole and Its Metabolite 5-hydroxyomeprazole** Omeprazole and its metabolite 5-hydroxyomeprazole concentrations in the plasma were determined by using a validated LC/MS-MS method. Prior to injection into the analytical system, isolation of omeprazole, its metabolite and the deuterated IS (omeprazole –d3) from 200 µL of human Li-heparin plasma was performed by solid phase extraction performed on Oasis HBL 60mg 3cc cartridges (Waters, Zellik, Belgium). The elution from the cartridges was done with 1 mL of methanol and then the eluate was evaporated to dryness under a stream of nitrogen at approximatively +35°C. The residue was reconstituted with 0.2 mL of mobile phase and then centrifuged at about 3500 rpm for 5 minutes at +5°C. 120 µL of the clear supernatant was transferred into injection vials. 10µL or 25 µL of the reconstituted sample was injected into the chromatographic system, depending on the LC/MS sensitivity. Chromatographic separation was performed on a Zorbax XBD-C8 column (Agilent, Eindhoven, The Netherlands) (50 x 4.6 mm, 3.5 µm from Interchim) by using a Nexera HLPC system (Shimadzu, Kyoto, Japan) in isocratic elution mode. The aqueous mobile phase consisted of a mixture of acetonitrile:water (25:75, v/v) containing 0.04% of ammonium hydroxide adjusted to pH 8.5 with formic acid. An API3000 mass spectrometer (AB Sciex, Nieuwerkerk aan den Ijssel, The Netherlands) equipped with a TIS probe operated in the MRM in negative mode was used for quantification. The precursor‐to‐product ion pairs at the mass‐to‐charge ratio (m/z) were 344.0 to 193.9, 359.9 to 149.1 and 347.1 to 197.0 for omeprazole, its metabolite and for the IS. The calibration curves in plasma for both compounds were linear over the range of 1–1000 ng/mL with 1/x^2^ as weighting factor. The limit of quantification of the assay in the plasma samples was set at 1 ng/mL for omeprazole and its metabolite.

**Caffeine and Its Metabolite Paraxanthine** Caffeine and its metabolite paraxanthine concentrations in the plasma were determined by using a validated LC/MS-MS method. Prior to injection into the analytical system, isolation of caffeine, paraxanthine and their respective deuterated IS (caffeine–d3 and paraxanthine–d3) from 100 µL of human Li-heparin plasma was performed by solid phase extraction performed on Oasis HBL 30 mg 1 cc cartridges (Waters, Zellik, Belgium). The elution from the cartridges was done with 0.8 mL of methanol and then the eluate was evaporated to dryness under a stream of nitrogen at approximatively at +35°C. The residue was reconstituted with 1.5 mL of 2 mM tridecafluoroheptanoic acid/acetonitrile mixture (80/20, v/v) and then transferred into injection vials. 10 µL of the reconstituted sample was injected into the chromatographic system. Chromatographic separation was performed on a Zorbax SB phenyl column (150 x 3 mm, 3.5 µm from Agilent, Eindhoven, The Netherlands) set at +40°C by using an HLPC system (Shimadzu, Kyoto, Japan). A gradient mobile phase was used, starting at 90% mobile phase A (2 mM tridecafluoroheptanoic acid) and 10% mobile phase B (acetonitrile) for the first 2 min, and then with a linear increase over 20 min to 20% mobile phase B, followed at 20.1 min by 4.9 min re-equilibration to initial condition. The total run time was 25 min. An API365 mass spectrometer (AB Sciex, Nieuwerkerk aan den Ijssel, The Netherlands) equipped with a TIS probe operated in the MRM in positive mode was used for quantification. The precursor‐to‐product ion pairs at the mass‐to‐charge ratio (m/z) were 195.2 to 138.0, 181.2 to 124.1, 198.3 to 137.9 and 184.2 to 127.1 for caffeine, its metabolite and their respective IS. The calibration curves in plasma for both compounds were linear over the range of 100–50 000 ng/mL with 1/x^2^ as weighting factor. The limit of quantification of the assay in the plasma samples was set at 100 ng/mL for caffeine and its metabolite.

**References**

1. Timmis H, Van Kaem T, Desrivot J, et al. GLPG1205, a GPR84 modulator: safety, pharmacokinetics, and pharmacodynamics in healthy subjects. *Clin Pharmacol Drug Dev.*  2021:in press DOI: 10.1002/cpdd.955

2. Zeilinger K, Freyer N, Damm G, Seehofer D, Knospel F. Cell sources for in vitro human liver cell culture models. *Exp Biol Med (Maywood).* 2016;241(15):1684-1698.

3. Bjornsson TD, Callaghan JT, Einolf HJ, et al. The conduct of in vitro and in vivo drug-drug interaction studies: a PhRMA perspective. *Journal of clinical pharmacology.* 2003;43(5):443-469.

4. Smith PK, Krohn RI, Hermanson GT, et al. Measurement of protein using bicinchoninic acid. *Analytical biochemistry.* 1985;150(1):76-85.

5. Kanebratt KP, Andersson TB. HepaRG cells as an in vitro model for evaluation of cytochrome P450 induction in humans. *Drug Metab Dispos.* 2008;36(1):137-145.

6. Easterbrook J, Lu C, Sakai Y, Li AP. Effects of organic solvents on the activities of cytochrome P450 isoforms, UDP-dependent glucuronyl transferase, and phenol sulfotransferase in human hepatocytes. *Drug metabolism and disposition: the biological fate of chemicals.* 2001;29(2):141-144.

7. Jia L, Liu X. The conduct of drug metabolism studies considered good practice (II): in vitro experiments. *Curr Drug Metab.* 2007;8(8):822-829.

8. Relling MV, Aoyama T, Gonzalez FJ, Meyer UA. Tolbutamide and mephenytoin hydroxylation by human cytochrome P450s in the CYP2C subfamily. *The Journal of pharmacology and experimental therapeutics.* 1990;252(1):442-447.

9. Tassaneeyakul W, Birkett DJ, Veronese ME, et al. Specificity of substrate and inhibitor probes for human cytochromes P450 1A1 and 1A2. *The Journal of pharmacology and experimental therapeutics.* 1993;265(1):401-407.

10. Wrighton SA, Stevens JC, Becker GW, VandenBranden M. Isolation and characterization of human liver cytochrome P450 2C19: correlation between 2C19 and S-mephenytoin 4'-hydroxylation. *Arch Biochem Biophys.* 1993;306(1):240-245.

**Supplementary Table 1.** Exclusion Criteria for Subjects in the Drug–Drug Interaction Study.

| **Exclusion Criteria** |
| --- |
| Poor or moderate metabolizer for CYP2C9 or CYP2C19 as determined by genotyping.* |
| Having a contraindication as indicated in the respective Summary of Product  Characteristics (or Package Leaflets) for warfarin, omeprazole or caffeine. |
| Intake of nutraceuticals (e.g., St. John’s wort) within 3 weeks prior to day 1 of treatment period 1 or within 6 times the elimination half-life of the neutraceutical, whichever was the longest. |
| Intake of enzyme inducing or enzyme inhibiting drugs within 3 months prior to day 1 of treatment period 1. |
| Intake of vitamin K within 3 weeks prior to day 1 of treatment period 1. |
| Known hypersensitivity to study drug ingredients or a significant allergic reaction to any drug as determined by the investigator, such as anaphylaxis requiring hospitalization. |
| Positive serology for hepatitis B virus surface antigen (HBsAg) or hepatitis C virus  (HCV) or any history of hepatitis from any cause with the exception of hepatitis A. |
| History of or a current immunosuppressive condition (e.g., human immunodeficiency virus [HIV] infection type 1 and 2). |
| Symptoms of clinically significant illness in the 3 months before the initial study drug administration. |
| Presence or having sequelae of gastrointestinal, liver or kidney (creatinine clearance ≤ 80 mL/min using the Cockcroft–Gault formula; if calculated result ≤ 80 mL/min, a 24-hour urine collection to determine actual value could be done) or other conditions known to interfere with the absorption, distribution, metabolism, or excretion of drugs. |
| History of malignancy within the past 5 years (except for basal cell carcinoma of the skin that has been treated with no evidence of recurrence). |
| Clinically relevant abnormalities detected on electrocardiogram regarding either rhythm or conduction (eg, QTcF ≥450 ms, or a known long QT syndrome). A first degree heart block or sinus arrhythmia was not considered as a significant abnormality. |
| Clinically relevant abnormalities detected on vital signs. |
| Significant blood loss (including blood donation [>450 mL]), or had a transfusion of any blood product within 12 weeks prior to the initial study drug administration. |
| Hemoglobin level below 12 g/dL. |
| Elevated prothrombin time as determined by prothrombin time (international normalized ratio) >1.3. |
| Treatment with any drug known to have a well-defined potential for toxicity to a major organ in the last 3 months preceding the initial study drug administration. |
| Active drug or alcohol abuse (more than 3 glasses of wine or beer or equivalent/day) within 2 years prior to the initial study drug administration. |
| Regular consumption of a large quantity of coffee, tea (>6 cups per day) or equivalent. |
| Administration of an injectable drug within 30 days prior to the initial study drug administration, with the exception of local anesthetics. |
| Concurrent participation, or participation within 8 weeks prior to the initial study drug administration in a drug/device study. |
| Investigator or any sub-investigator, research assistant, pharmacist, study coordinator, or other staff or relative thereof who was directly involved in the conduct of the study. |
| Any condition or circumstances that in the opinion of the investigator could make a  subject unlikely or unable to complete the study or comply with study procedures and requirements. |
| Current sexually active (and/or child wish) male that did not agree to use a contraception method from the time of first dose of study drug, during the study and until 12 weeks after the last study drug dose. |

*Responsible for screen failure in 16 out of 39 subjects.

**Supplementary Table 2.** Phenacetin O-deethylase Activity (CYP1A2) in Cultured Human Hepatocytes.

| Compound | Batch HEP220793 | | Batch HEP220794 | | | Batch HEP220795 | | |
| --- | --- | --- | --- | --- | --- | --- | --- | --- |
|  | Fold induction | % of positive control | | Fold induction | % of positive control | | Fold induction | % of positive control |
| Control (0.1% DMSO) | 1.00 | - | | 1.00 | - | | 1.00 | - |
| Omeprazole 50 µM | 53.9 ±5.00 | 100 | | 10.7 ±0.6 | 100 | | 35.2 ±1.8 | 100 |
| Control (0.4% DMSO) | 1.00 | - | | 1.00 | - | | 1.00 | - |
| GLPG1205 0.1 µM | 1.64 ± 0.256 | 1.22 | | 0.929 ± 0.049 | –0.73 | | 1.22 ± 0.099 | 0.656 |
| GLPG1205 0.5 µM | 1.86 ± 0.197 | 1.63 | | 0.878 ± 0.047 | –1.26 | | 1.45 ± 0.107 | 1.33 |
| GLPG1205 1 µM | 2.24 ± 0.152 | 2.35 | | 0.933 ± 0.086 | –0.69 | | 1.59 ± 0.158 | 1.71 |
| GLPG1205 5 µM | 1.96 ± 0.021 | 1.81 | | 0.802 ± 0.052 | –2.04 | | 1.41 ± 0.123 | 1.20 |
| GLPG1205 10 µM | 1.00 ± 0.036 | 0.00 | | 0.469 ± 0.076 | –5.46 | | 0.640 ± 0.080 | –1.05 |
| GLPG1205 50 µM | 0.335 ± 0.033 | –1.26 | | 0.367 ± 0.021 | –6.51 | | 0.335 ± 0.036 | –1.95 |

DMSO, dimethylsulfoxide

**Supplementary Table 3.** CYP1A2 mRNA Expression in Cultured Human Hepatocytes.

| Compound | Batch HEP220793 | | Batch HEP220794 | | | Batch HEP220795 | | |
| --- | --- | --- | --- | --- | --- | --- | --- | --- |
|  | Fold induction, mean (SD) | % of positive control | | Fold induction, mean (SD) | % of positive control | | Fold induction, mean (SD) | % of positive control |
| Control (0.1% DMSO) | 1.00 ± 0.11 | - | | 1.00 ± 0.24 | - | | 1.00 ± 0.26 | - |
| Omeprazole 50 µM | 341 ± 88.1 | 100 | | 403 ± 30.8 | 100 | | 85.9 ± 9.74 | 100 |
| Control (0.4% DMSO) | 1.00 ± 0.16 | - | | 1.00 ± 0.30 | - | | 1.00 ± 0.25 | - |
| GLPG1205 0.1 µM | 2.97 ± 0.43 | 0.58 | | 0.57± 0.12 | –0.11 | | 1.59 ± 0.36 | 0.69 |
| GLPG1205 0.5 µM | 3.67 ± 0.26 | 0.79 | | 0.66 ± 0.12 | –0.09 | | nd | nd |
| GLPG1205 1 µM | 6.12 ± 0.65 | 1.51 | | 0.73 ± 0.26 | –0.07 | | 4.51 ± 0.02 | 4.13 |
| GLPG1205 5 µM | 5.33 ± 0.90 | 1.27 | | 1.60 ± 0.45 | 0.15 | | 3.53 ± 0.64 | 2.98 |
| GLPG1205 10 µM | 3.28 ± 0.20 | 0.67 | | 0.50 ± 0.04 | –0.12 | | 1.18 ± 0.33 | 0.22 |
| GLPG1205 50 µM | 1.58 ± 0.12 | 0.17 | | 1.15 ± 0.72 | 0.04 | | 0.62 ± 0.05 | -0.44 |

DMSO, dimethylsulfoxide; nd: not determined; SD, standard deviation

**Supplementary Figure 1.** Chemical structure of GLPG1205 (9-cyclopropylethynyl-2-((S)-1-[1,4]dioxan-2-ylmethoxy)-6,7-dihydropyrimido[6,1-a]isoquinolin-4-one; compound code G321605).

**
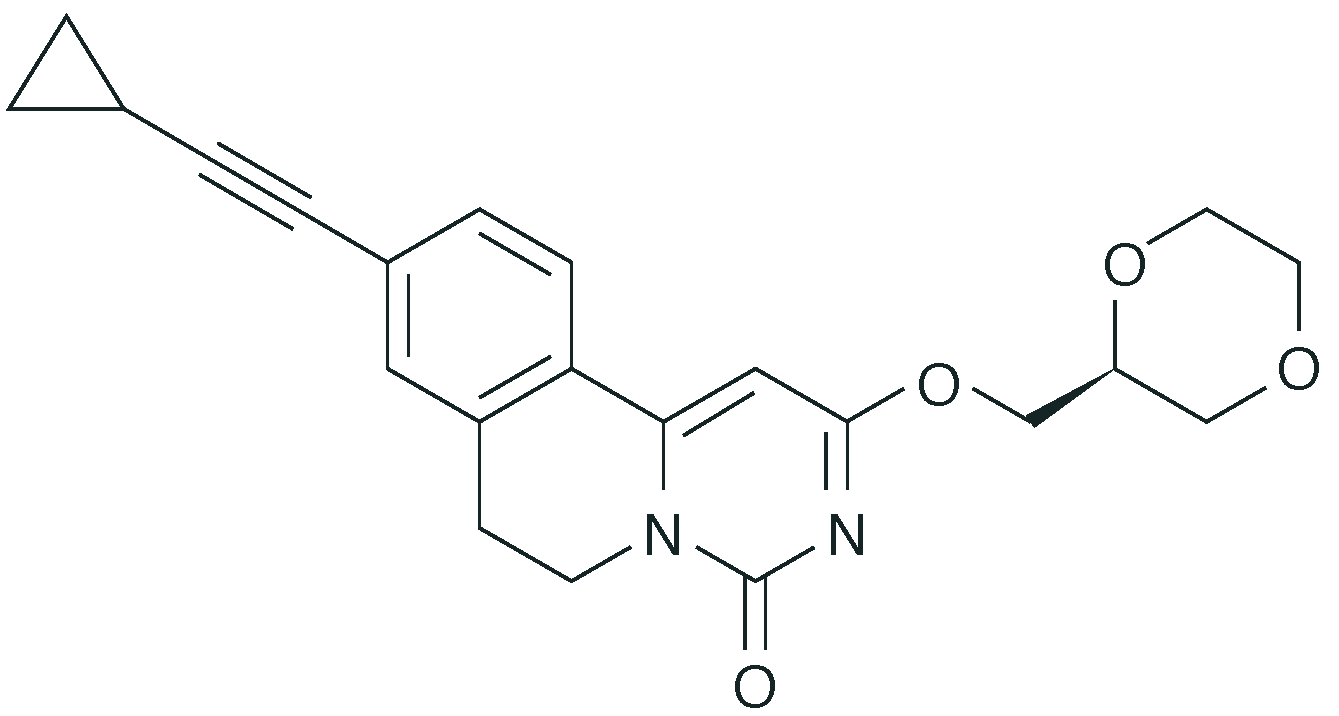
**

**Supplementary Figure 2.** Effects of GLPG1205 0.5–100 µM on the concentration of hydroxytolbutamide (CYP2C9 substrate) in human liver microsomes. Additional incubations were performed with: 1) a 30-minute preincubation step of GLPG1205 with microsomes before addition of the cofactor NADPH and CYP2C9; and 2) a 30-minute preincubation step of GLPG1205 with microsomes and NADPH prior to the addition of CYP2C9. Data are percentage of vehicle control ± standard deviation.


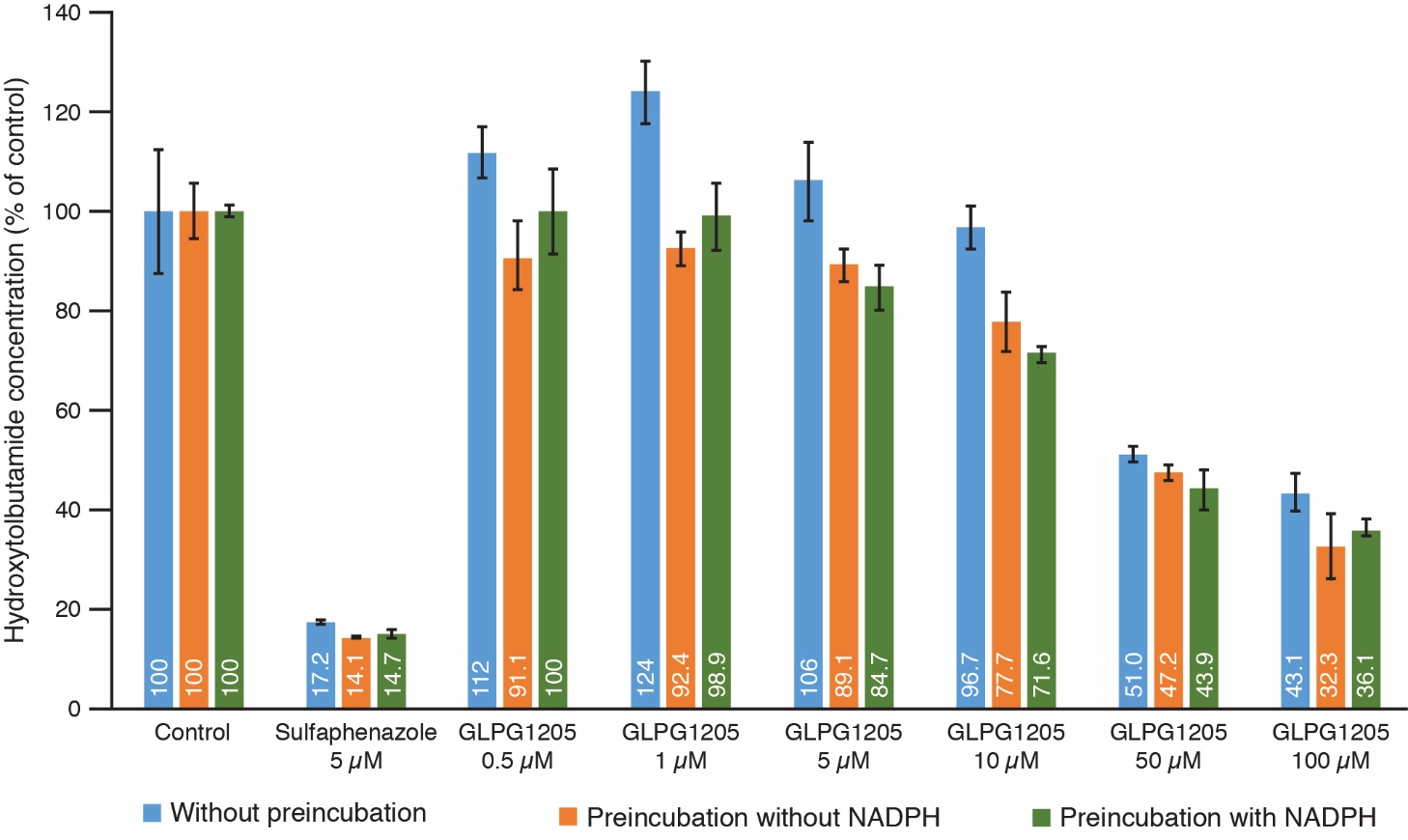


**Supplementary Figure 3** Effects of GLPG1205 0.5–100 µM on the concentration of hydroxymephenytoin (CYP2C19 substrate) in human liver microsomes. Additional incubations were performed with: 1) a 30-minute preincubation step of GLPG1205 with microsomes before addition of the cofactor NADPH and CYP2C19; and 2) a 30-minute preincubation step of GLPG1205 with microsomes and NADPH prior to the addition of CYP2C19. Control 1: 1% methanol; control 2: 0.5% methanol + 0.5% DMSO. Data are percentage of vehicle control ± standard deviation.


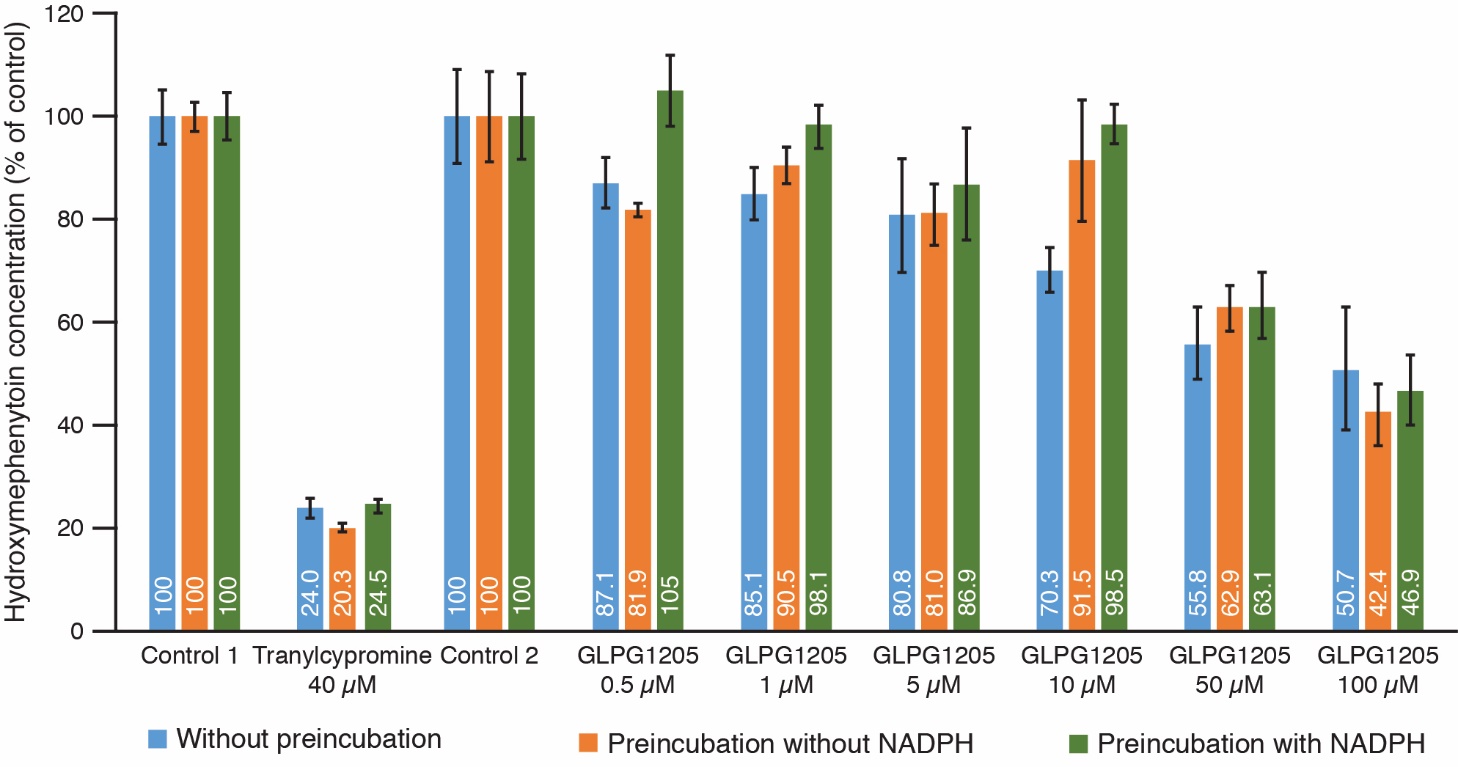


**Supplementary Figure 4** Effects of GLPG1205 0.5–100 µM on the concentration of acetaminophen (CYP1A2 substrate) in human liver microsomes. Additional incubations were performed with: 1) a 30-minute preincubation step of GLPG1205 with microsomes before addition of the cofactor NADPH and CYP1A2; and 2) a 30-minute preincubation step of GLPG1205 with microsomes and NADPH prior to the addition of CYP1A2. Data are percentage of vehicle control ± standard deviation. *Acetaminophen was below the limit of quantification for Furafylline/preincubation with NADPH.


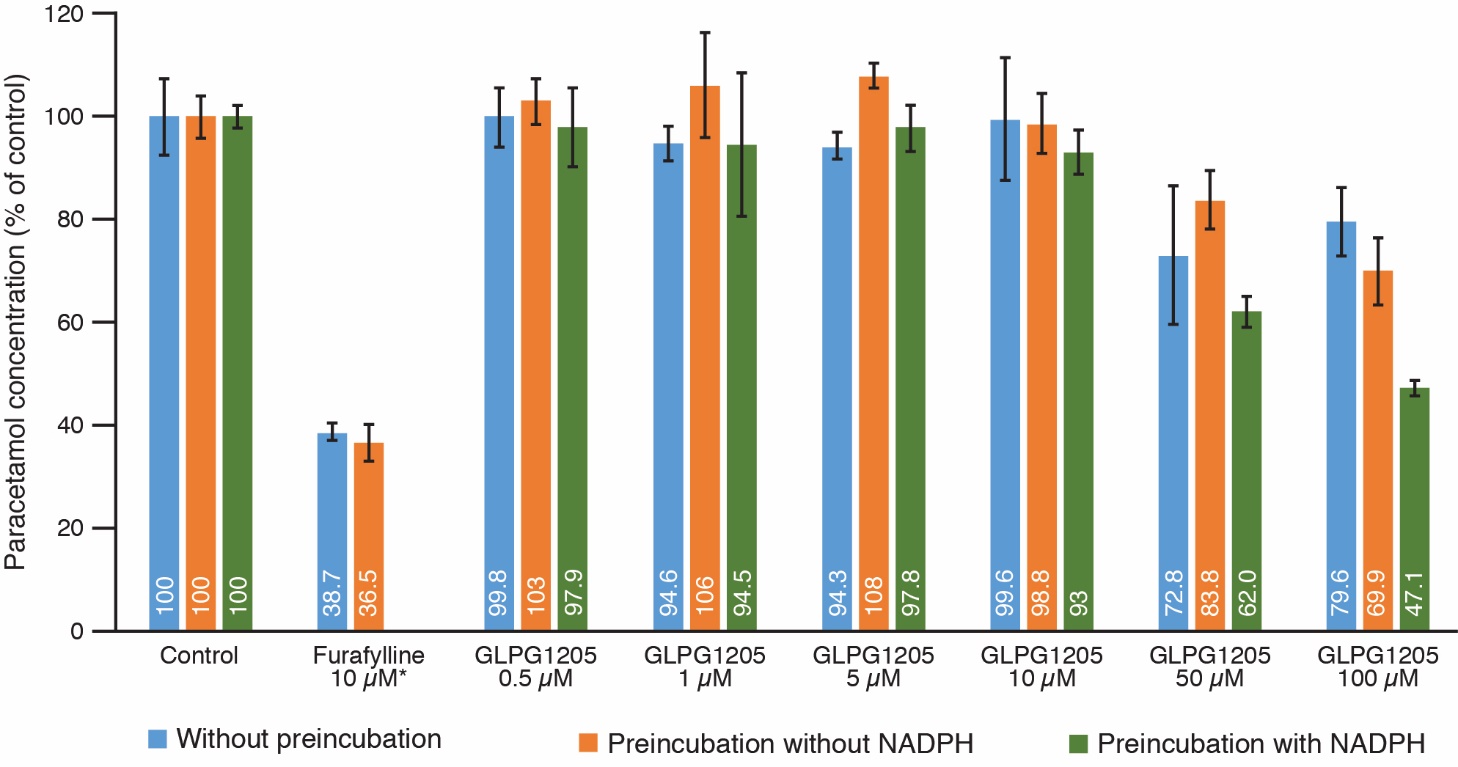

Supplement: Supplementary file 1 — Supplemental Information [file CPDD-10-1007-s001.docx]
